# Return to sport after total knee arthroplasty: an Australian perspective

**DOI:** 10.1007/s00402-025-05843-7

**Published:** 2025-04-11

**Authors:** Mahsa Sarrami, George Awwad, David Parker

**Affiliations:** 1 Royal North Shore Hospital, St Leonards, Australia; 2 University of Sydney, Sydney, Australia; 3 Sydney Orthopaedic Research Institute, St Leonards, Australia; 4 Flinders Medical Centre, Bedford Park, Australia; 5 Level 1, The Gallery, 445 Victoria Avenue, Chatswood, NSW 2067 Australia

**Keywords:** Total knee replacement, Total knee arthroplasty, Return to sport

## Abstract

**Purpose:**

Total knee arthroplasty is a well-established procedure that aims to improve pain, mobility and function. Multiple studies have demonstrated a strong correlation between patient satisfaction and their preoperative expectations, including return to sport. This study aims to assess the level of post-operative activity and rate of return to sport after TKA in an Australian population.

**Methods:**

A retrospective electronic survey was distributed amongst patients that were 1–3 years post total knee arthroplasty. Patient characteristics and sport participation pre and post operatively was assessed. Standardised questionnaires including University of California at Los Angeles (UCLA) activity-level scale and Forgotten Joint Score (FJS) were conducted to evaluate activity level and subjective functional outcomes. Further questions regarding barriers to return to sport were distributed.

**Results:**

257 individuals (128 male, 129 female), with mean age of 68 participated in our study. Highest rate of return to pre-operative sport was found in walking, yoga, swimming and bushwalking with an overall rate of 81% return to pre-operative sport. Statistically significant increase was observed in UCLA activity-level score 1.14 and FJS score increase of 54.63. Barriers to return to sport included medical co-morbidities, loss of interest, fear of damage to the implant, and stiffness and pain in the operated knee.

**Conclusion:**

A high rate of return to preoperative sport can be achieved after TKA, but can still probably be enhanced further with more focused rehabilitation specific to individual patient expectations.

**Level of evidence:**

III.

## What are the new findings

Data on Australian population showing increased return to sport post total knee arthroplasty.

Return to surfing post total knee arthroplasty is common in Australian population.

## Introduction

Broader indications for total knee arthroplasty (TKA) and improved patient outcomes and implant survivorship are resulting in higher numbers of younger and more active patients undergoing surgery [1, 2]. Satisfaction from post-operative outcome is shown to be highly correlated with preoperative expectations, with evolving expectations in both younger and older populations to remain active [3, 4]. Thereby, a commonly encountered discussion between patients and clinicians is the expectations around return to activity and sport post TKA.

A systematic review and meta-analysis of 25 studies, demonstrated a median return to sport (RTS) rate of 71.2% [5]. Across the studies there was a variation in the definition of RTS, making comparisons difficult, and many studies were limited by small participant numbers. Common sports and activities studied included walking, cycling and swimming. Different populations have different demographics, expectations and common sports [6]. The data predominantly reports on low impact activity and there is a paucity of data on wider variety of sports participated by the general population, including no data found on surfing [7].

The purpose of this study is to evaluate return to sport and recreational activity after TKA in the Australian population, in order to provide relevant recommendations to inform surgeon and patient expectations.

## Methods

We performed a retrospective review of prospectively collected demographic data conducted for TKA from March 2018–2020 at a single centre (Sydney Orthopaedic Research Institute, Socrates database). All patients underwent a TKA by one of four consultant orthopaedic surgeons at two private hospitals in Sydney, Australia. All included patients were followed-up at a minimum of one year postoperatively. Revision TKA and unicompartmental knee arthroplasty were excluded.

The preoperative demographics and patient reported outcome measurement scores (PROMS) were collected prospectively as part of routine data collection. Further questionnaire was developed for this study to collect specific information regarding pre-operative and post-operative sport participation in a retrospective format. This was distributed to participants via email and phone calls. The pre-operative time period was defined as the 12 months prior to the surgery. The 15 sports with the greatest participation rates by the Australian adult population were included in the questionnaire, in addition to skiing. Recreational walking is the most common sport participated by the Australian population [8]. The questionnaire differentiated between walking for activities of daily living and recreational walking for health and exercise, with the former not considered as an activity for this study. Preoperative sporting participation was specified as activity during the 12 months timeframe leading to the surgery. Patient perception of reasons for failed return to their chosen sport was also assessed. University of California at Los Angeles (UCLA) activity scale and Forgotten Joint Scores (FJS) were collected for every patient.

Ethics approval was obtained from the Northern Sydney Local Health District Human Research Ethics Committee (2019/ETH08340). Informed consent was obtained from each patient for the use of their information and outcomes for research purposes.

Power analysis was conducted to determine the sample size for the total population size of 585 patients in order to gain a significant 1 score difference for UCLA as the outcome measure. It generated a required sample size of 232 patients to achieve a 95% confidence level with 0.05 confidence interval. Further statistical analysis was performed using *jamovi* (Version 1.6.23.0; Australia, 2021). Patient demographics were described using means and standard deviation. Descriptive analyses were performed to describe sport activity and compare UCLA and FJS scores pre and post operatively. Significance level was set as *p* < 0.05. Further analysis using a two-tailed student’s t-test was used for continuous scores.

## Results

585 patients were contacted during the study period. There were 257 respondents and 336 total knee arthroplasties, all of which were included in the study (Table 1). Gender distribution was equal (males: *n* = 128, females: *n* = 129), with a mean age of 68 years (range 49–87, SD ± 7.2) and mean BMI of 29 (SD ± 5.3). Of the study cohort, 79 (31%) of patients underwent bilateral TKA and 178 (69%) underwent unilateral TKA.

**Table 1:** Demographics of 257 participants at time of TKA

	*N*=	Mean (SD)
Age (years)	257	68 (7.2)
Sex		
Males	128	
Females	129	
BMI	223	29.6 (5.25)
Bilateral	79	
Unilateral	178	
Right	107	
Left	71	

63% of participants identified sport as a relevant part of their life. UCLA activity score increased by a mean of 1.074 (95% CI 0.836–1.312; *p* < 0.005) compared to pre-operative scores. This was statistically significant for all age groups. Post-operation means for UCLA score decreased with increasing age. Mean FJS increased by 49.56 (95% CI 21.847–32.713; *p* < 0.005) compared to pre-operative score. This was also statistically significant for all age groups. Post-operative mean FJS increased with increasing age.

A trend can be observed between different age categories and UCLA scores and FJS (Table 2). For both UCLA scores and FJS, the lowest pre-operative mean and greatest mean differences are observed in the younger population groups.

**Table 2:** UCLA activity score and FJS pre and post operatively and comparison of different age groups

	*N*=	Pre-operation mean	Post-operation mean	Mean difference (95% CI)	*P*-value
UCLA					
49–59	27	4.704	6.556	1.852 (1.078–2.625)	< 0.005
60–69	115	5.217	6.461	1.244 (0.870–1.617)	< 0.005
70–79	102	5.637	6.324	0.687 (0.328–1.045)	< 0.005
80–89	13	4.769	5.769	1 (0.111–1.889)	0.031
All	257	5.307	6.381	1.074 (0.836–1.312)	< 0.005
FJS					
49–59	14	9.376	61.458	52.082 (36.257–67.908)	< 0.005
60–69	70	13.594	62.26	48.668 (40.696–56.640)	< 0.005
70–79	65	14.821	65.417	50.596 (43.473–57.719)	< 0.005
80–89	8	21.356	65.885	44.529 (36.257–67.908)	0.012
All	157	14.121	63.681	49.56 (21.847–32.713)	< 0.005

Further sub-analysis of our population to review young population (< 55 years) performance compared to general population aged over 55 years (Table 3). Showed greater mean difference and lower pre-operative scores in younger patients for outcome scores of both UCLA and FJS.

**Table 3:** UCLA activity score and FJS pre and post operatively comparing young patients vs. general population

	Age	*N*=	Pre-operation mean	Post-operation mean	Mean difference (95% CI)	*P*-value
FJS	≤ 55	12	33.333	69.618	36.285 (12.84–59.73)	0.0058
	56+	245	36.583	65.408	28.825 (23.372–34.278)	< 0.005
UCLA	≤ 55	12	4	6.167	2.167 (0.991–3.342)	< 0.005
	56+	245	5.371	6.392	1.02 (0.778–1.263)	< 0.005

Further review of participation of the young population defined as less than 55 showed variable return rates as compared to general population (Table 4). On average each individual under 55 years pre-operatively participated in 5.4 different types of sport compared to 4.2 for the population 56 or older. Post operatively this rate remained higher in the younger population (4.8 compared to 4).

**Table 4:** Sport participation pre and post operatively of young patient (≤ 55) vs. general population (56+)

	Participants 55 or younger (*n* = 12)			Participants 56 or older (*n* = 245)		
	Pre-operative participation	Post-operative participation	% change	Pre-operative participation	Post-operative participation	% change
Walking	10	11	110%	217	234	108%
Swimming	8	5	63%	146	141	97%
Gym	9	10	111%	133	129	97%
Bushwalking	5	6	120%	109	114	105%
Cycling	9	7	78%	79	79	100%
Golf	1	0	0%	77	69	90%
Surfing	3	1	33%	45	45	100%
Pilates	2	1	50%	45	33	73%
Yoga	3	5	167%	24	25	104%
Skiing	1	1	100%	23	18	78%
Tennis	4	3	75%	30	16	53%
Athletic	2	2	100%	17	10	59%
Soccer	1	1	100%	7	5	71%
AFL	0	0	NA	5	4	80%
Basketball	1	0	0%	2	2	100%
Netball	0	0	NA	2	4	200%
Other	6	5	83%	74	44	59%
Total sum of sports	65	58	89%	1035	972	94%
Average number of Sport per individual	5.4	4.8	90%	4.2	4	94%

Table 5 illustrates the number of individuals that returned to the sport or activity they participated in pre-operatively. Highest rates were seen in walking (98%) and yoga (92%). Lowest rates were seen in higher impact sports such as of basketball, athletics, AFL and tennis.

Overall rate of return to any activity postoperatively was 98% across all patients. Individual sporting participation pre and postoperatively demonstrated an individual rate of return to the same pre-operative sport of 81%.

**Table 5:** Number and percentage return of individuals that returned post-operatively to the same sport as their pre-operative participation

	Returned to pre-operative sport (*n*)	% of return
Walking (recreational)	224	98
Yoga	25	92
Swimming	136	88
Bushwalking	96	84
Gym	116	81
Golf	63	80
Cycling	69	78
Surfing	34	71
Skiing	17	70
Pilates	31	65
Soccer	5	62
Netball	1	50
Tennis	15	44
AFL	2	40
Athletic	7	36
Basketball	1	33
Other	41	58

Most common new sports included recreational walking, gym, bushwalking and cycling (Table 6). The commonest new activity post-operatively was recreational walking, with 70% of nonparticipants taking up the activity post-TKA. Patients have modified what activities they do post operatively. Overall, 34% of all individuals participated in at least one new sport post operatively.

**Table 6:** Frequency of participation in a new sport post-operatively

	New participants post-TKA *n*=	Non-participants pre-TKA *N*=	New sport post-TKA (%)
Walking (recreational)	21	30	70
Gym	23	115	20
Bushwalking	24	143	16
Cycling	17	169	10
Swimming	10	103	9
Surfing	12	209	5
Golf	6	179	3
Yoga	5	230	2
Athletic	5	238	2
Tennis	4	223	1
Netball	3	255	1
Pilates	3	210	1
Skiing	2	233	0
AFL	2	252	0
Soccer	1	249	0
Basketball	1	254	0
Other	10	187	5

The most common reason to not return to sport was other health related problems (7.68%) and loss of interest (7%) (Table 7). Only 8% of participants reported specific issues of stiffness and / or pain in their TKA as a factor limiting their activities.

**Table 7:** Reasons participants attributed to not returning to some of their sports

Reasons why participants did not return to sport	%
Other health problems	7.68
Lost interest	7.04
Fear of damage to operated knee	6.4
Stiffness in operated knee	5.12
Pain in operated knee	2.88
Surgeon advice	2.88
Not applicable - I returned to the sports I was playing	68.1

**Fig. 1:**
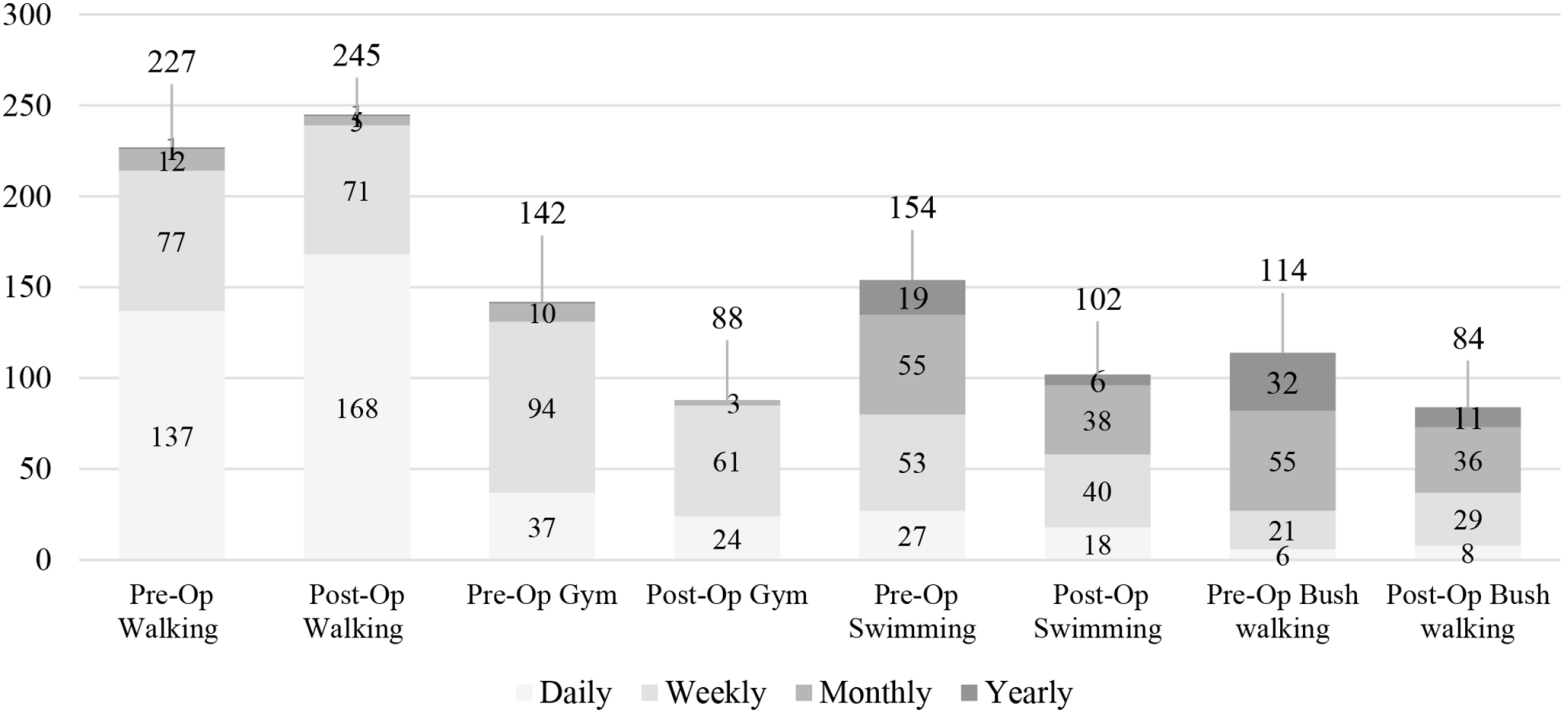
Frequency of participation in sports Pre-Operatively compared to Post-operatively

Further review of the sports with over 100 pre-operatively participation was done to review frequency of participation in each sport (Fig. 1). Recreational walking not only showed increase in overall participation, but also 31 further individuals participated on a daily basis.

## Discussion

Total knee arthroplasty is an increasingly common procedure performed to improve quality of life, largely through the relief of pain and improvement of mobility. Reported rates of dissatisfaction post TKA are often high [9], and meeting a patient’s preoperative expectations has been shown to be a major factor in achieving patient satisfaction [10]. Increasingly, for many patients the ability to return to sport and regular exercise is a significant goal.

It is difficult to interpret the literature when discussing return to sport rates after TKA. Variations in definitions of return to sport, sporting participation across differing geographical regions, and differing patient baseline demographics are all factors. Previous studies have demonstrated RTS rates are influenced by regional and geographical factors. For example mountainous countries report increased participation in skiing due to greater accessibility [11–13]. The geographical differences in Australia have their own implications on the distribution of specific participation relevant to the population, such as surfing.

Hanriech et al. conducted a systematic review of 25 studies investigating return to sport post TKA, reporting a median RTS rate of 71.2% [5]. Their review found wide discrepancies in the definition of RTS participation and timing. Jassim et al. [14] quoted a return to athletic activity as 100%, however, considered any return to activity as a successful return to sport. A similar definition was used by Bock et al. [15], identifying a RTS rate of 89.2%. In our study, only 5 individuals did not participate in any activity postoperatively, with 98% of all individuals thus returning to a level of activity postoperatively. To better enable clinicians to counsel postoperative expectations, we utilised a definition tailored to individual sport and considered preoperative sporting engagement. Return to sport in our study was defined as post-operative return to a sport participated by individuals pre-operatively. The overall rate of RTS was 81%. Cognisant of the limitations of sport-specific rates of RTS in the literature, further studies are required to counsel expectations for our patients.

There were significant variations in rates of return to specific sports, as demonstrated in Table 3. This ranged from a 33% return rate for basketball and 98% for recreational walking. The pattern of our results is consistent with other literature demonstrating higher return rates to lower impact activities such as recreational walking, yoga, swimming and bushwalking [5]. Other high impact sports such as AFL (Australian Rules Football), netball, and soccer had lower return rates, but were also limited by smaller sample sizes [5].

### Sport specific rates of return to sport

#### Recreational walking

The most participated activity in the adult Australian population is recreational walking (46%) [8]. In our population 98% of individuals who participated preoperatively were able to return to recreational walking post operatively. Of the individuals (*n* = 30) who were not pre-operatively participating in recreational walking, 70% (*n* = 21) were able to do so post-=peratively. This trend was similarly seen by Chatterji et al., with increased participation post-operatively [7]. This high level of return is reflected in the population motivation and signifies the importance of participating in this activity for our population group.

#### Surfing

Nearly one in ten Australians surf recreationally [16], however despite its prevalence in Australia there are no studies reporting the outcomes of surfing following TKA. This presumably relates to a sport such as surfing being considered as unrealistic post TKR, demonstrating the need to update these studies to more contemporary patient expectations. The novel nature of our large Australian cohort identified that 48 individuals surfed leading up to surgery with 34 (71%) of these individuals returned to surfing post operatively. 12 (5%) individuals who did not surf leading up to surgery were able to surf post-operatively.

#### Golf

Jackson et al. [17], reviewed 93 golfers and found 81% reported ongoing participation post total knee replacement. Our study reviewed the general population and found the same return rate of 81% (63 from 78). A further 6 individuals picked up the sport without any participation leading up to the surgery. Other studies compared variable rates of return between TKA and unicompartmental knee arthroplasty with return to golf in 30% and 100% respectively [18], although this difference may reflect differing patient populations rather than a direct effect of implant choice.

#### Tennis

34 individuals participated in tennis prior to TKA with 15 (44%) returning postoperatively. Four individuals engaged with tennis as a new sport postoperatively. Our study shows an increased return to tennis compared to rates quoted by other studies (0–33%) [7, 15, 19]. Neither our study nor the quoted studies report on participation in a single vs. double or social vs. competitive space. Previous surveys have commonly reported orthopaedic surgeon recommendations for social-level doubles tennis [20].

### Patient reported outcome scores

UCLA activity score and FJS showed statistically significant improvements postoperatively. When reviewing age-adjusted UCLA and FJS scores, the younger cohorts demonstrated the largest improvements. This is consistent with the meta-analysis by Hanriech et al., with improvements to UCLA activity scores across all age groups, and the greatest improvements observed in the younger population [5]. This finding may be influenced by variations in motivation to return to activity and baseline demographic risk [21–24]. FJS similarly demonstrated greater score improvements in younger population groups, consistent with other studies [25].

Patient factors affect outcomes in participation of sports. When asked a generic question of ‘is sport relevant to your life?’ only 63% of our participants said yes. This illustrates a mixed cohort with different expectations and goals. Almost half the patients that did not return to their chosen sport attributed it to loss of interest and other health related problems. Huch et al. found that caution and pain elsewhere were the most common reasons for reduction in sport participation [19].

In our cohort, most common reason for not returning to sport was either related to other health problems or loss of interest. This shows that the majority of our cohort decided to reduce or change their sporting preferences based on reasons other than the outcome of the TKA (stiffness and pain). Other studies have shown that one of the main contributors to not returning is surgeon advice [14, 26]. Although this represented a small group of individuals for our cohort it is an important one to consider, as the impact of surgeon advice can be tailored more specifically on a case by case basis with more informed discussions. This variation in patient interest and expectations can provide clinicians with targeted and individualised rehabilitation goals for motivated individuals whom sport participation remains a goal.

Whilst a rate of return to sport can be useful, frequency of participation can provide further insight into individual outcomes. Increased sporting frequency after TKA may present as a surrogate for improvement, whereas a reduction in frequency is also impacted by confidence, interest and accessibility. Thus, the quality of performance or patient satisfaction from participation of each individual sport may more significantly reflect a successful return to sport.

### Limitations and future implications

Limitations of this study include the risk of selection bias and recall bias associated with the retrospective nature of the study. From 585 identified potential patients, 257 (44%) responded to our survey, with a natural risk of selection bias with participants willing to respond to questionnaires being more optimistic or of a more active population. Our method of data collection relied on use of online technology, which can limit participation of some individuals and contribute to loss of follow-up. Accounting for other variables that impact return to sport such as baseline risk, motivation and accessibility and other comorbidities can be difficult. Furthermore, a single centre study are inherently at risk of selection bias due to demographics and environmental factors that may not represent the wider and regional communities. Moreover, postoperative rehabilitation was not internally protocolled, with rehab programs thus being heterogenous and not dedicated to patient expectations for a return to individual sports.

Whilst patients are able to return to sport post TKA, defining “sport” and what constitutes a successful return is another challenge. Further studies evaluating individual sports and the quality of return to each sport would enable targeted strategies on improving participation and satisfaction. The accuracy of the data from patients is not objective sporting participation as detailed level of return to sport was not analysed. Other strategies to improve rate of return to sport would be to counsel patients both pre and post-operatively and engage them in sport specific rehab goals and programs.

## Conclusion

The current study provides a review of a large Australian cohort with an overall high rate of return to sport post total knee arthroplasty, including unique sports such as surfing. This study can be used to inform individual patient counselling on expectations in participation in sports post TKA.
